# Early Awake Prone Position Combined with High-Flow Nasal Oxygen Therapy in Severe COVID-19: A Case Series

**DOI:** 10.1007/s44231-022-00026-z

**Published:** 2022-12-22

**Authors:** Y. Chengfen, Z. Yongle, L. Jianguo, X. Lei

**Affiliations:** 1 The Third Central Hospital of Tianjin, Tianjin, 300170 China; 2 Tianjin Key Laboratory of Extracorporeal Life Support for Critical Diseases, Artificial Cell Engineering Technology Research Center, Tianjin Institute of Hepatobiliary Disease, Tianjin, China; 3 Tianjin Haihe Hospital, Tianjin, 300170 China

**Keywords:** Awake prone position, High-flow nasal oxygen therapy, COVID-19, SARS-CoV-2

## Abstract

**Background:**

Awake prone positioning has been used for non-intubated patients with COVID-19-related acute hypoxaemic respiratory failure, but the results are contradictory. We aimed to highlight the role of awake prone positioning combined with high-flow nasal oxygen therapy in severe COVID-19 patients infected with the Delta variant of SARS-CoV-2.

**Methods:**

From June 12 to December 7, 2021, we successfully performed prone position(PP) combined with high-flow nasal oxygen(HFNO) therapy on two patients infected with the delta variant of SARS-CoV-2. HFNO was prescribed to reach SpO_2_ ≥ 92%. PP was proposed to patients with PaO_2_/FiO_2_(P/F) < 150 mmHg. Arterial blood gas (ABG) and hemodynamic were monitored before and after PP sessions. The target time of PP was more than 12 h per day and could be appropriately shortened according to the patient’s tolerance. Relevant clinical data, HFNO parameters, PICCO parameters, P/F ratio and PP duration were obtained from medical records.

**Results:**

A total of 23 PP sessions and 6 PP sessions combined with HFNO were performed in case 1 and case 2, respectively. Compared with values before PP, GEDI, ELWI and Qs/Qt decreased significantly (GEDI: 869.50 ± 60.50 ml/m^2^ vs. 756.86 ± 88.25 ml/m^2^; ELWI: 13.64 ± 2.82 ml/kg vs. 12.43 ± 2.50 ml/kg; Qs/Qt: 15.32 ± 6.52% vs. 12.24 ± 5.39%; all p < 0.05), Meanwhile, the oxygenation improved significantly (P/F: 184.50 ± 51.92 mmHg vs. 234.21 ± 88.84 mmHg, p < 0.05), The chest CT revealed the lung infiltrates improved significantly after PP. Both cases were discharged to a dedicated COVID-19 ward without requiring intubation.

**Conclusions:**

Combining PP with HFNO could be a useful treatment strategy for avoiding intubation in severe COVID-19 patients infected with the Delta variant of SARS-CoV-2 to improve pulmonary vascular involvement, improve oxygenation and avoid intubation, but further studies are needed to validate our approach.

## Background

Since the first report of cases from India in late 2020, the delta variant of SARS-CoV-2(DVSC-2) has become the predominant strain in much of the world [1]. Awake prone positioning has been used for non-intubated patients with COVID-19-related acute hypoxaemic respiratory failure, but the results are contradictory. We aimed to highlight the role of awake prone positioning (PP) combined with high-flow nasal oxygen therapy (HFNO) in severe COVID-19 patients infected with the Delta variant of SARS-CoV-2.

## Case Presentation

**Fig. 1:**
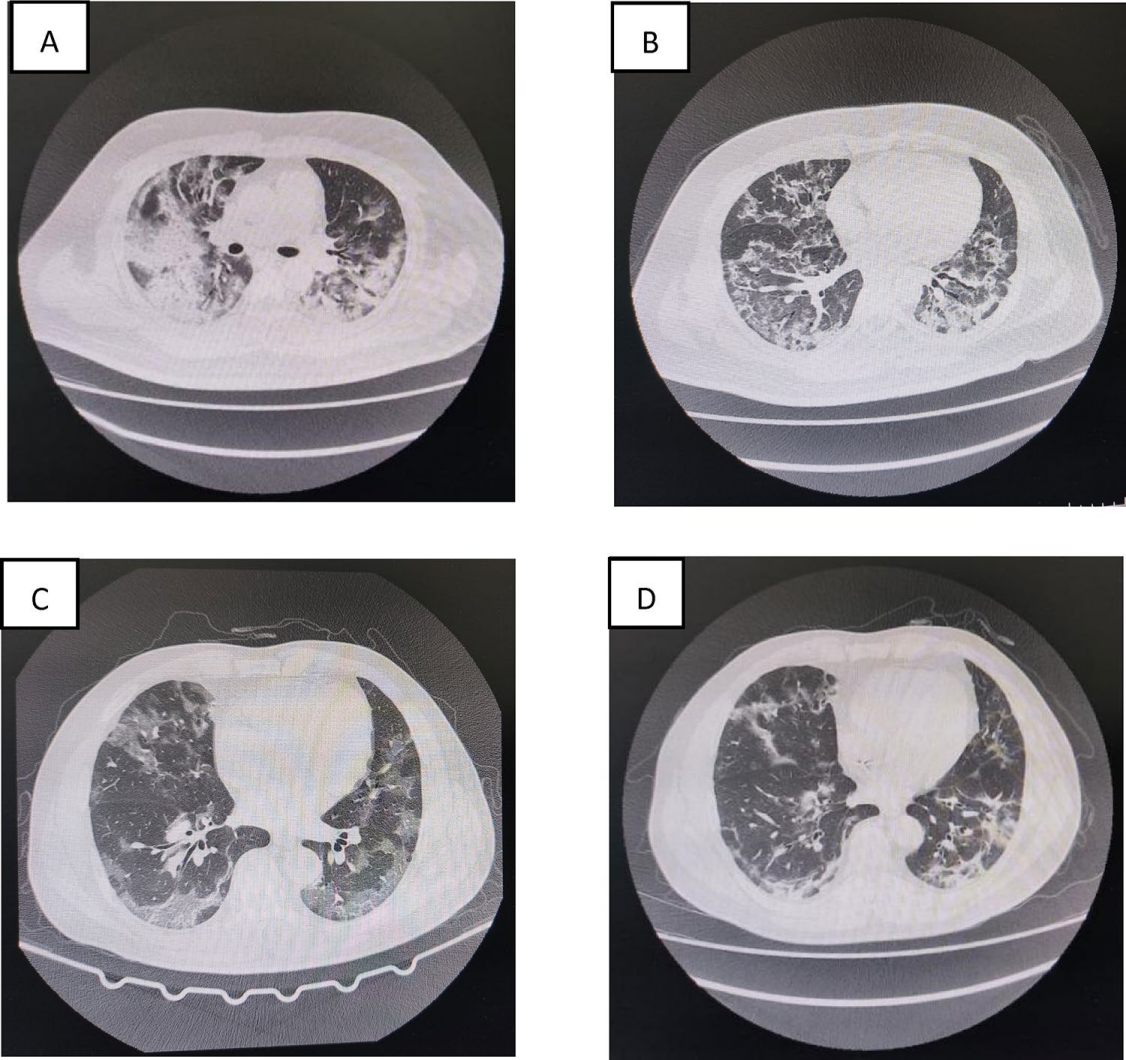
Chest computed tomographic scans in the two severe COVID-19 patients infected with the delta variant of SARS-CoV-2. Chest computed tomographic (CT) scan taken before prone position (PP) in case 1. **B** CT scan taken on after PP in case 1. It revealed the lung infiltrates improved significantly compared with **A**. **C** CT scan taken before PP in case 2. **D** CT scan taken after PP in case 2. It revealed the lung infiltrates improved significantly compared with **D**

From June 12 to December 7, 2021, 80 COVID-19 patients mainly infected with DVSC-2 were screened. Two of them had whole genome sequencing-confirmed DVSC-2 infection. They were graded as severe according to the Guidelines for the Diagnosis and Treatment of Novel Coronavirus (2019-nCoV) Infection by the National Health Commission (trial version 8). Both patients presented with bilateral opacities suggestive of pneumonia that rapidly worsened after several days of admission (Fig. 1). We successfully performed PP combined with HFNO therapy on them.

HFNO was prescribed to reach SpO_2_ ≥ 92%. PP was proposed to patients with PaO_2_/FiO_2_(P/F) < 150 mmHg. The PICCO was proposed for hemodynamic monitoring and volume management on their admission to intensive care unit (ICU). Arterial blood gases (ABG) and hemodynamics were monitored before and after PP sessions. The target time of PP was more than 12 h per day and could be appropriately shortened according to the patient’s tolerance. Relevant clinical data, HFNO parameters, PICCO parameters, P/F ratio and PP duration were obtained from medical records and are presented in Table 1.

**Table 1:** Clinical characteristics of patients

Case no	Case 1	Case 2		
Gender	Male	Male		
Age(years)	44	44		
BMI (kg m^−2^)	28.9	26.7		

Comorbidity	Pharyngitis rhinitis hypertension diabetes														Mean ± SD	P value
Sessions	1	2	3	4	5	6	7	8	9	10	11	12	13	14		
HFNO parameter, Flow (l/min)/FiO_2_ (%)	50/55	50/55	50/55	50/55	50/50	50/50	50/50	45/40	45/40	35/29	50/50	45/45	45/45	40/40		
Before PP																
CI (l/min/m^2^)	3.25	3.17	3.32	3.12	2.74	2.64	2.75	3.03	2.81	3.71	3.59	3.19	3.91	3.52	3.20 ± 0.39	
SVRI (dyn.s.cm^−5^ m^2^)	2401	2472	2419	2329	2526	2544	2526	2275	2419	2512	2768	2979	2633	2324	2509.07 ± 187.24	
CVP (mmHg)	5	5	5	7	3	3	5	4	4	2	4	10	13	5	5.36 ± 2.93	
GEDI (ml/m^2^)	885	897	794	949	793	799	917	811	935	923	937	875	868	790	869.50 ± 60.50	
ELWI (ml/kg)	19	14	12	13	16	115	14	15	11	17	15	10	9	11	13.64 ± 2.82	
MAP (mmHg)	109	96	88	75	81	75	73	81	82	89	74	94	70	98	84.64 ± 11.44	
HR (bpm)	88	90	96	92	78	68	80	78	84	72	78	104	80	88	84.00 ± 9.67	
RR (breath/min)	26	24	22	26	21	20	21	22	20	20	19	20	17	22	21.42 ± 2.53	
Qs/Qt (%)	31.23	23.69	17.96	16.75	15.89	17.56	14.36	7.65	10.37	7.89	18.36	13.20	10.62	8.96	15.32 ± 6.52	
PaO_2_ (mmHg)	54.4	74.1	87.7	77.3	91.9	79.7	86.3	83.8	74.5	61.7	69.7	111	116	112	84.30 ± 18.49	
PaO_2_/FiO_2_ (mmHg)	99	135	159	141	184	159	173	210	186	213	139	247	258	280	184.50 ± 51.92	
After PP																
CI (l/min/m^2^)	3.24	3.4	3.34	3.15	3.12	2.78	2.62	2.93	2.74	3.25	3.43	2.88	4.14	3.95	3.21 ± 0.44	0.82
SVRI (dyn.s.cm^−5^ m^2^)	2408	2383	2419	2347	2383	2454	2687	2347	2454	2528	2537	28,833	2668	2268	2483.29 ± 165.34	0.35
CVP (mmHg)	5	3	3	7	3	3	4	4	4	2	4	10	13	5	5.00 ± 3.06	0.10
GEDI (ml/m^2^)	824	701	761	842	594	672	786	653	936	824	740	802	749	712	756.86 ± 88.25	0.00
ELWI (ml/kg)	17	13	12	13	14	12	15	13	12	13	14	7	10	9	12.43 ± 2.50	0.02
MAP (mmHg)	102	87	65	76	68	80	74	92	94	76	94	84	87	97	84.00 ± 11.21	0.85
HR (bpm)	100	104	98	80	70	64	78	80	78	76	76	88	84	84	82.86 ± 11.36	0.62
RR (breath/min)	27	25	24	21	21	20	21	23	23	20	17	20	16	20	21.29 ± 2.95	0.79
Qs/Qt (%)	22.32	15.36	18.63	17.31	16.78	16.32	10.59	6.59	6.98	5.24	10.35	8.71	8.69	7.51	12.24 ± 5.39	0.00
PaO_2_ (mmHg)	80.4	105	82	75	80.6	81	91.9	85.3	107	82	149	168	176	134	106.94 ± 35.12	0.01
PaO_2_/FiO_2_ (mmHg)	146	181	149	136	161	162	184	213	267	283	298	373	391	335	234.21 ± 88.84	0.01
Duration of prone positioning	13	13	11	13.5	13.5	6	12	11.5	11.5	13	8	9.5	8.5	7.5	10.82 ± 2.49	

A total of 23 PP sessions and 6 PP sessions combined with HFNO were performed in case 1 and case 2, respectively. ABG and hemodynamic were monitored before and after PP sessions during 10 PP sessions of case 1 and 4 PP sessions of case 2. The hemodynamic results showed their cardiac index (CI), systemic vascular resistance index (SVRI) and central venous pressure (CVP) were within normal range, however, global end-diastolic volume index (GEDI), intrapulmonary shunt fraction (Qs/Qt) and extravascular lung water index (ELWI)were higher than normal before PP. There was no volume responsiveness confirmed by passive leg rising test in both cases. Although their volume was limited, GEDI and ELWI were higher than normal. Compared with values before PP, GEDI, ELWI and Qs/Qt decreased significantly (GEDI: 869.50 ± 60.50 ml/m^2^ vs. 756.86 ± 88.25 ml/m^2^; ELWI: 13.64 ± 2.82 ml/kg vs. 12.43 ± 2.50 ml/kg; Qs/Qt: 15.32 ± 6.52% vs. 12.24 ± 5.39%; all p < 0.05), Meanwhile, the oxygenation improved significantly (PaO_2_: 84.30 ± 18.49 mmHg vs. 106.94 ± 35.12 mmHg; P/F: 184.50 ± 51.92 mmHg vs. 234.21 ± 88.84 mmHg, both p < 0.05), Nevertheless, there were no significant differences in respiratory rate (RR), heart rate (HR) and mean arterial pressure (MAP) between before and after PP (all p > 0.05). The chest CT revealed the lung infiltrates improved significantly after PP (Fig. 1). Both cases were discharged to a dedicated COVID-19 ward without requiring intubation. we used the paired t test for numerical variables to compare variables between before awake prone position and after awake prone position.

To our knowledge, this is the first description of severe COVID-19 patients infected with DVSC-2 managed effectively and improved pulmonary vascular involvement by PP combined with HFNO. Despres C et al. [2] showed similar findings in severe COVID-19 patients infected with the original version of SARS-CoV-2. People infected with DVSC-2 produce far more virus, acts on the angiotensin-converting enzyme 2 (ACE2), than do those infected with the original version of SARS-CoV-2 [1]. Alongside emerging understanding of the role of the ACE2 pathway in the pathogenic process, it has become clear that at least some COVID-19 patients, exhibit significant pulmonary vascular involvement [3, 4]. Besides volume overload and inflammation response, pulmonary vascular involvement caused by DVSC-2 might be one reason for elevated GEDI. Previous studies have confirmed that PP recruited some collapsed pulmonary microvessels through increasing central blood volume and decrease PVR and RV afterload through increasing the lung volume [5]. HFNO create a positive end expiratory pressure effect, contributing to decreasing the work of breathing and enhance oxygenation [6].

## Conclusion

Combining PP with HFNO could be a useful treatment strategy for avoiding intubation in severe COVID-19 patients infected with the Delta variant of SARS-CoV-2 to improve pulmonary vascular involvement, improve oxygenation and avoid intubation, but further studies are needed to validate our approach.

## Data Availability

Not applicable.

## Abbreviations

COVID-19: Coronavirus disease 2019
PP: Prone position
HFNO: High-flow nasal cannula
DVSC-2: Delta variant of SARS-CoV-2
PF: PaO_2_/FiO_2_
ACE2: The angiotensin-converting enzyme 2
